# OCT angiography analysis of choriocapillaris vascular density in different stages of age-related macular degeneration

**DOI:** 10.3389/fopht.2022.985262

**Published:** 2022-09-22

**Authors:** Maria Cristina Savastano, Claudia Fossataro, Matteo Mario Carlà, Chiara Fantozzi, Benedetto Falsini, Alfonso Savastano, Clara Rizzo, Raphael Kilian, Stanislao Rizzo

**Affiliations:** ^1^ Ophthalmology Unit, Fondazione Policlinico A. Gemelli, IRCCS, Rome, Italy; ^2^ Ophthalmology Unit, Università Cattolica del Sacro Cuore, Rome, Italy; ^3^ Ophthalmology Unit, University of Verona, Verona, Italy; ^4^ Consiglio Nazionale della Ricerca (CNR), Istituto di Neuroscienze, Pisa, Italy

**Keywords:** age-related macular degeneration, advanced exudative AMD, exudative AMD, geographic atrophy AMD, macular neovascularization, OCT angiography, choriocapillaris vascular density, disciform scar AMD

## Abstract

**Objectives:**

To analyze the choriocapillaris vessel density (CVD) of eyes at different stages of Age-related Macular Degeneration (AMD) with Optical Coherence Tomography Angiography (OCTA).

**Methods:**

This is a prospective observational cross-sectional study on 21 age-matched healthy eyes and 84 eyes with AMD (i.e., early AMD, late AMD, Geographic Atrophy [GA], and disciform scar AMD). OCTA was used to automatically measure the CVD (%), on both the whole macula and the foveal area, in a layer going from 9 µm above to 30 µm below the Bruch’s membrane. Furthermore, in the GA subgroup, the extension of the Ellipsoid Zone (EZ) interruption and the area of macular chorio-retinal atrophy was analyzed.

**Results:**

Macular CVD was significantly lower in the GA, late AMD and disciform scar AMD-subgroups compared to controls (respectively, p=0.0052; p<0.0001; p=0.0003), whereas it did not significantly vary in the early AMD group (p=0.86). A significant difference between the early AMD and both the late AMD and the disciform scar AMD subgroups was also found (p=0.0009 and 0.0095, respectively). When comparing the foveal CVD of healthy and AMD eyes, a significant difference was found with every AMD subgroup (early AMD, p=0.011; GA, p<0.0001; late AMD, p<0.0001; disciform scar AMD, p<0.0001). Furthermore, in the GA subgroup, the CVD had an inverse correlation with both the extension of the EZ-interruption (p=0.012) and with the calculated chorio-retinal atrophic area (p=0.009).

**Conclusions:**

OCTA could play a crucial role in the categorization of AMD, allowing for the evaluation of gradual flow impairment at different stages of the disease. Moreover, the detection of a decreased macular and foveal CVD may shed light on the pathogenesis of AMD.

## Introduction

Age-related macular degeneration (AMD) is the most prevalent cause of permanent visual loss in the elderly (1). The recent introduction of Optical Coherence Tomography Angiography (OCTA) has allowed to deepen our understanding of the retinal vasculature and its pathological changes, shedding light on the pathogenesis of many retinal vascular diseases, such as AMD. By Employing OCTA, retinal specialists are able to detect choroidal neo-vascularizations (CNVs), and to analyze their architecture as well as their response to anti vascular endothelial growth factor (anti-VEGF) intravitreal injections (2–5).

Several metabolic factors and cytokines, whose release seems to be promoted by an increased distress of the retinal pigment epithelium (RPE), are involved in the origin of macular neovascularization (MNV) in AMD (6). The role of the choroid and particularly the choriocapillaris (CC) in the pathophysiology of AMD is still controversial. From clinical and histological investigations, it seems like choroidal changes and choroidal vascular impairment are linked to the emergence and progression of both dry and exudative AMD (7, 8). Even though these vascular modifications have been clearly implicated in geographic atrophy (9–12), evidence of their correlation with the onset of MNV are still limited. Particularly, there is still debate on whether the origin of AMD has to be found in the RPE or in the CC (7). Nonetheless, what has already been demonstrated, is a decrease in CC blood flow with aging. The latter is probably due to repeated oxidative stress leading to retinal ischemia. The sequence of these events appears to play a major role in the origin of AMD (8–10).

With the advent of OCTA, CC blood flow can be studied in detail in order to understand its role in the pathogenesis of different forms of AMD. OCTA findings are even more relevant when considering that it is actually the onset of neovascularization (i.e., neovascular exudative AMD - NE-AMD) to be the most challenging issue to be faced in MNV patients. Friedman, who provided the first hemodynamic model of AMD, hypothesized that scleral stiffening could be the cause of an impaired choroidal blood flow, which in turn resulted in RPE damage and drusen production (13). Indeed, previews angiographic and histopathologic investigations have already shown a reduced choroidal blood flow in AMD eyes (14–17). Moreover, supporting the idea of macular hypoperfusion, recent studies have reported lower choriocapillaris density on OCTA across a whole range of AMD phenotypes (18–24). Choriocapillaris loss has also already been linked to drusen and geographic atrophy (GA) progression (12, 25, 26).

The aim of the study was to evaluate the choriocapillaris vessel density (CVD) of eyes at different stages of AMD and to compare it with sex- and age-matched healthy controls, in order to understand how choriocapillaris vascular network is affected along the progression of the disease. The secondary aim was to assess the possible correlation between the size of macular chorio-retinal atrophy in patients affected by dry AMD and the decrease in CVD.

## Materials and methods

We conducted a prospective observational cross-sectional study including 5 subgroups that were classified as follows: healthy controls over 70 years of age, early – AMD, Geographic Atrophy (GA), late AMD under treatment with anti-VEGF intravitreal injections, and disciform scar AMD.

The study was conducted at the Fondazione Policlinico Universitario Agostino Gemelli, IRCCS, in Rome, Italy from September 2021 to January 2022. The study adhered to the tenets of the Declaration of Helsinki and was approved by Catholic University of the Sacred Heart Ethical Committee in Rome, Italy (ID number: 3860). An informed and written consent was obtained from all enrolled patients. All participants received a comprehensive ophthalmic examination which included the measurement of the best corrected visual acuity (BCVA) according to the Early Treatment for Diabetic Retinopathy Study (ETDRS) (BCVA was converted into LogMAR scale for statistical analysis), slit-lamp biomicroscopy, a dilated funduscopic examination, SD-OCT (Spectral Domain – Optical Coherence Tomography) and OCTA (Solix full – range OCT, Optovue Inc, Freemont CA, USA).

### Patients

This study included patients affected by dry (non-exudative) or wet (exudative) AMD (d-AMD and w-AMD, respectively) and a healthy control group. Exclusion criteria were clinically significant lens opacities, SD-OCT or OCTA scans’ signal quality less than 7/10, systemic illnesses with ocular involvement, previous pars plana vitrectomy (PPV), concomitant vascular retinal diseases, congenital eye disorders, uveitis and myopia or hyperopia greater than 3.0 diopters (D).

Based on ophthalmoscopic and imaging examination, patients were classified as follows:

A) Healthy over-70 years of age group: healthy patients aged more than 70 years with no objective retinal diseases.
B) Stage I and II age-related maculopathy group (early AMD group): patients with confirmed diagnosis of AMD and possible mild vision loss. Fundoscopy and imaging evaluation may show medium-sized drusen (≥ 63 - <125 μm), in the absence of pigmentary abnormalities (stage I) or one large druse n (≥ 125 μm) with possible pigmentary abnormalities (stage II) (27).
C) Geographic Atrophy (GA) group: patients at late stage of macular degeneration and severe vision loss showing on fundoscopy and imaging evaluation a localized sharply demarcated atrophy of the outer retinal tissue layers, retinal pigment epithelium and choriocapillaris.
D) Late AMD group: patients who had developed an MNV associated with signs of activity (i.e., intra-retinal, sub-retinal or sub-RPE fluid, dark halo, branching of MNV), still undergoing anti-VEGF intravitreal injections. According to clinical indication, we used the specific on label anti-VEGF agent, choosing between Bevacizumab, Ranibizumab, Aflibercept and Brolucizumab;
E) Disciform scar AMD group: patients with a previous history of chronic exudative AMD that evolved into a disciform scar due to retinal fibrosis. These patients had already suspended anti-VEGF intravitreal injections.

Inclusion criteria in each subgroup were evaluated by three expert ophthalmologists independently. If all three operators agreed, the patient was consequently assigned to a specific subgroup. Cohen coefficient analysis was used to ensure that the concordance was at least 0.90. Otherwise, the experts discussed the case and if a univocal choice could not be reached, the patient was excluded from the study.

Ultimately, in a *post-hoc* analysis we included a supplementary group of 21 healthy controls less than 70 years of age, to also assess whether a correlation between CVD and age may exist, even in healthy eyes.

### Imaging protocol and acquired data

OCT and OCTA scans were performed using the Solix full-range OCT (Optovue Inc, Freemont, CA, USA), a new ultra-high-speed spectral domain device (version 2019 V1.0.0.317) that runs at 120.000 A-scans per second and uses the split spectrum amplitude-decorrelation angiography (SSADA) algorithm. Performing multiple B-scans in sequence, the software detects changes between each single scan which permits to identify the vasculature (28).

Before performing the exams, each eye was dilated with 1% Tropicamide drops. High-definition line B scans, passing horizontally and vertically through the fovea were obtained, as well as a fundus photography. The OCTA scan consisted of a 6.4x6.4 mm image focused on the fovea. The software adopts Motion Correction Technology, a unique post-processing technology that allows for genuine three-dimensional (3D) distortion correction in all directions for ultra-precise motion correction. Scans with a low-quality index (≤ 7/10) (e.g., significant lens opacities, frequent eye blinks, excessive movement artifacts) were rejected. Retinal features on OCT scans were evaluated to define the stage of AMD.

**Figure 1:**
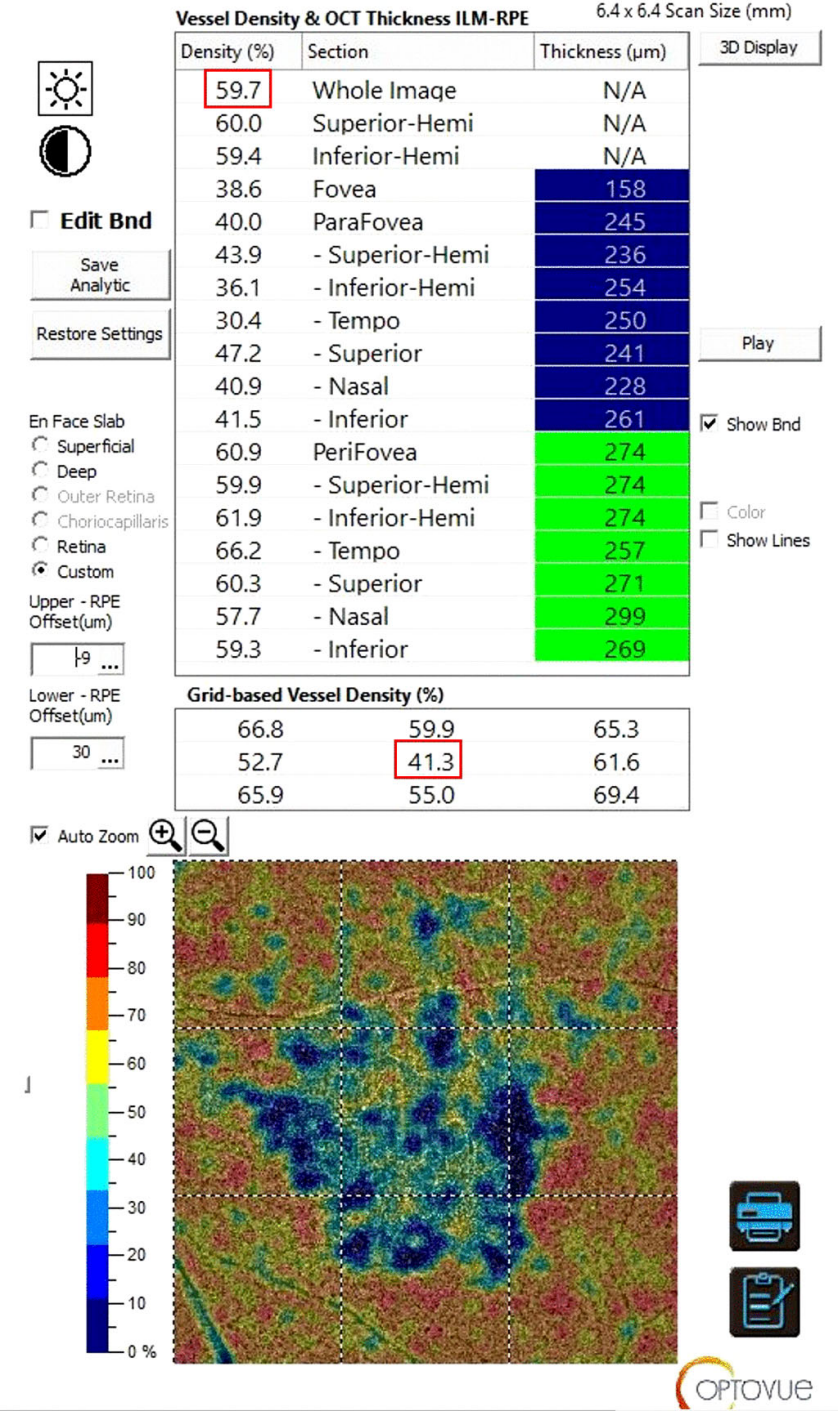
Sample of a Solix full-range OCTA analysis of the CVD using the in-built software algorithm. After setting a customized layer from 9 µm above to 30 µm below the Bruch’s Membrane the CVD of the *whole* macular area (highlighted by the upper red rectangle) and of the foveal only area (in the *fovea grid-based* image, highlighted by the lower red rectangle*)*, expressed in percentage, were collected. OCTA, Optical Coherence Tomography Angiography; CVD, Choriocapillaris Vascular Density.

The CVD of the *whole* macular area and of the foveal area (in the *fovea grid-based* image*)*, expressed in percentage, were analyzed in a customized layer set from 9 µm above to 30 µm below the Bruch’s Membrane, and calculated using the in-built software algorithm, as showed in **Figure 1**. Before processing the OCTA scans, two operators independently evaluated if the Bruch’s Membrane was correctly identified by the software. If the segmentation was not precise, the operator would have marked the layer manually.

**Figure 2:**
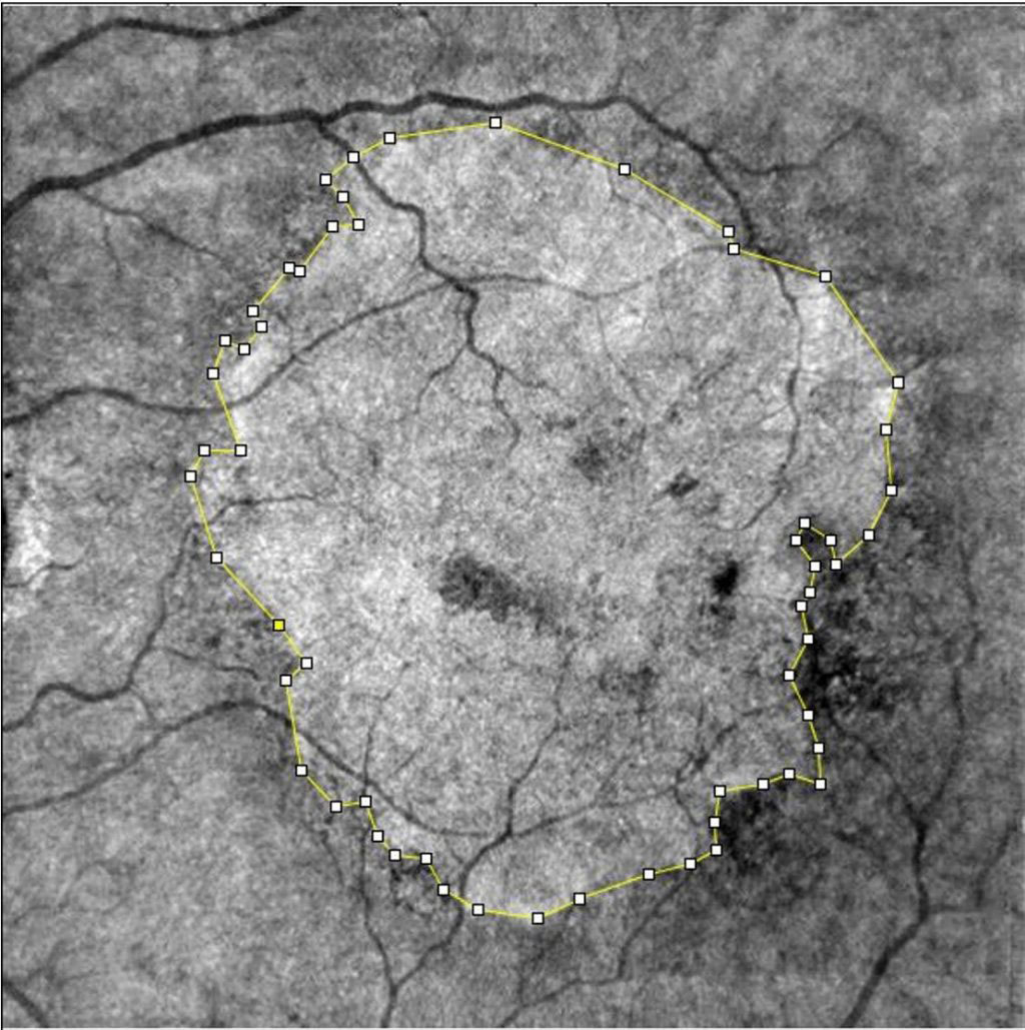
Sample of manual segmentation of a GA through ImageJ software tool. En-face images were exported from Solix full-range OCTA, after automatic segmentation at the retinal RPE. GA, Geographic Atrophy; OCTA, Optical Coherence Tomography Angiography; RPE, Retinal Pigmented Epithelium.

Additional analyses were conducted in the GA subgroup. Fovea-centered 6.4 mm horizontal B scans were collected in order to manually evaluate the extension (µm) of the Ellipsoid Zone (EZ) interruption *via* the built-in linear caliper tool. Additionally, we estimated the area of macular chorio-retinal atrophy, using the in-built image capture option for en-face 6.4x6.4 mm scans segmented at the RPE layer. Every scan capture was uploaded on ImageJ software (ImageJ 1.53k, National Institutes of Health, USA) and underwent an 8-bit conversion. Subsequently, the atrophic area was manually segmented and after putting the scale setting on 6.4x6.4 mm, the final value of the atrophic area (mm^2^) was calculated with the specific software tool (**Figure 2**).

### Statistical analysis

The sample size of each group was evaluated using the G-Power software package (Version 3.1.9.6). Assuming a minimum difference of 15%, a residual standard deviation of 10%, a power of 0.08 and an alpha of 0.05 to highlight the differences, the required smallest population size was 21 patients for each group. GraphPad PRISM Software (Version 9.0; GraphPad, La Jolla, CA) was used to analyze the resulting data. Shapiro-Wilk test was used to assess the normality of our sample, with *p value* > 0.05 set to verify the null hypothesis. We conducted an Analysis of Variance (ANOVA) and employed the Dunnett’s multiple comparison test to evaluate the differences in CVD amongst the various groups. Furthermore, a Tukey test, computing confidence intervals, was used to compare the difference between each pair of means. Finally, correlation and regression analyses were conducted for continuous variables. Quantitative values were expressed as mean ± SD and a p value <0.05 was considered statistically significant. A designated confidence interval (CI) of 95% was used.

## Results

**Figure 3:**
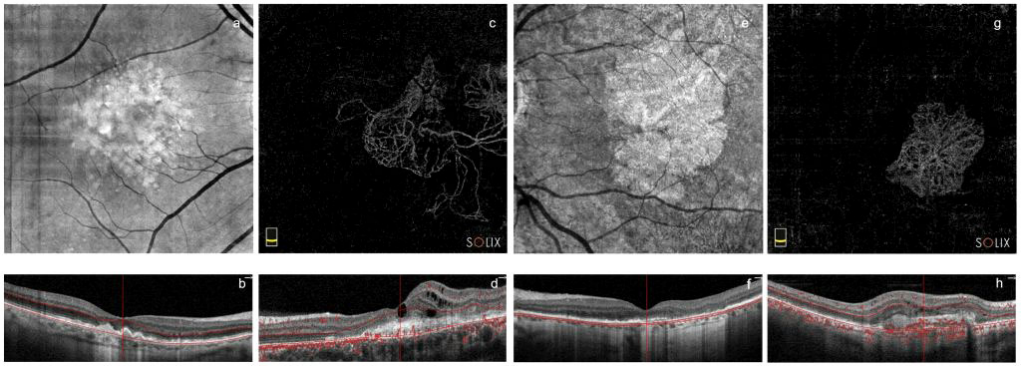
Sample images of every pathological subgroup from Solix full-range OCTA captures. En-face and B-scan images of early AMD group (**A, B**, respectively) and of the GA group (**E, F**, respectively). Outer retinal OCTA en-face images and B-scans showing MNVs and blood flow (red dots in B-scan images) in late AMD (**C, D**, respectively) and disciform scar AMD (**G, H**, respectively) groups. OCTA, Optical Coherence Tomography Angiography; MNVs, Macular Neo-Vascularizations; GA, Geographic Atrophy; AMD, age related macular degeneration.

Hundred-and-five eyes of 105 patients were examined, 21 for each subgroup. Demographic characteristics of all subgroups are listed in **Table 1**. OCTA samples are shown in **Figure 3**.

**Table 1:** Demographic characteristics of all sub-groups.

Mean data ( ± SD)	Healthy > 70	Early AMD	GA	Late AMD	Disciform Scar AMD
**Patients (n)**	21	21	21	21	21
**Age (yo)**	76.0 ± 5.8	73.4 ± 6.7	78.2 ± 7.7	78.0 ± 5.7	76.0 ± 7.5
**Gender (F/M)**	14/7	14/7	15/6	9/12	12/9
**BCVA (LogMAR)**	0.05 ± 0.09	0.10 ± 0.10	0.92 ± 0.76	0.87 ± 0.65	1.56 ± 0.12

GA, Geographic Atrophy; AMD, age related macular degeneration.

### Whole image CVD results

**Figure 4:**
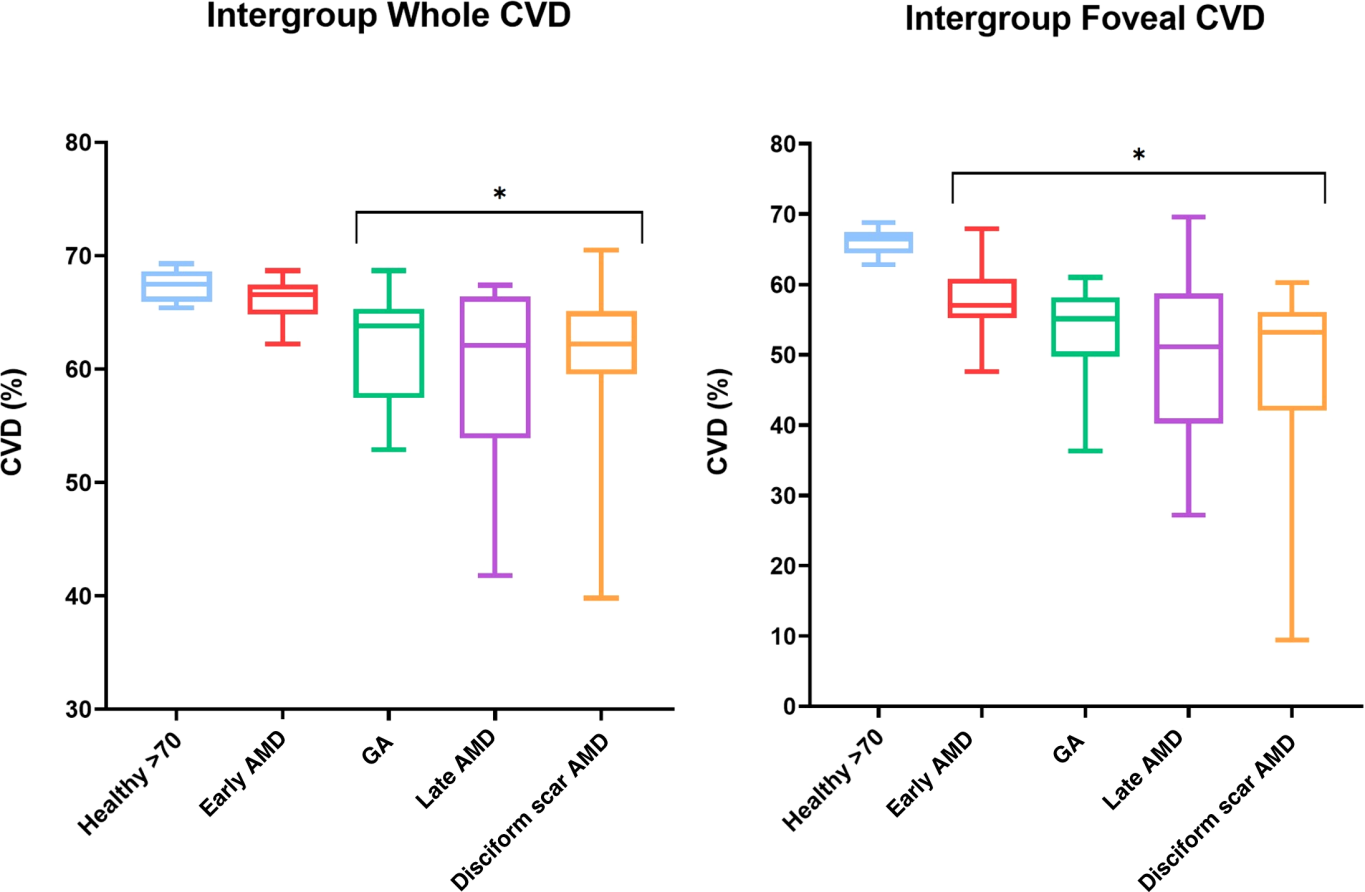
Box and whiskers graphs showing both whole macular and foveal area CVD variations among subgroups with min-to-max values. Asterisks* highlighting statistically significant differences when compared with healthy over 70 years-old subjects. CVD, Choriocapillaris Vascular Density; GA, Geographic Atrophy; AMD, age related macular degeneration.

There was a statistically significant difference between the *whole image*-CVDs (i.e., entire macular area) of the exudative, geographic atrophy and disciform scar AMD groups, and the control group (p <0.001). Multiple comparison analysis showed statistically significant differences as follows: Healthy vs. GA (mean difference 5.44, CI 1.33 to 9.54, p=0.0052); Healthy vs. late AMD (mean difference 8.01, CI 3.90 to 12.10, p <0.0001); Healthy vs. disciform scar AMD (mean difference 6.83, CI 2.72 to 10.90, p=0.0003). No statistically significant difference was found in the Healthy vs. early AMD group comparison (mean difference 1.26, CI -2.85 to 5.36, p=0.86) (**Figure 4**). The *post hoc* Tukey test showed statistically significant differences between early AMD and both late and disciform scar AMD groups (mean differences 6.75 and 5.57, CI 2.34 to 11.16 and 1.16 to 9.80, with p values to be 0.0009 and 0.0095, respectively). None of the other pairwise comparisons among subgroups showed statistically significant differences.

### Foveal grid-based CVD results

The ANOVA test confirmed a statistically significant difference between healthy and AMD groups (p <0.0001). Multiple comparison analyses showed statistical validity when comparing Healthy controls to all of the pathological subgroups: Healthy vs. early AMD (mean difference 7.80, CI 1.46 to 14.14, p=0.011); Healthy vs. GA (mean difference 13.20, CI 6.85 to 19.54, p<0.0001); Healthy vs. late AMD (mean difference 16.40, CI 10.05 to 22.74, p<0.0001); Healthy vs. disciform scar AMD (mean difference: 17.07, CI 10.72 to 23.41, p<0.0001) (**Figure 4**). Tukey’s multiple comparisons test was conducted among all pathological subgroups, showing no statistically significant difference among the pathologic subgroups, with the exception of the early AMD vs. late AMD and disciform scar AMD comparison (mean differences 8.60 and 9.27, CI 1.81 to 15.39 and 2.48 to 16.06, with p=0.0095 and p=0.0041, respectively).

**Figure 5:**
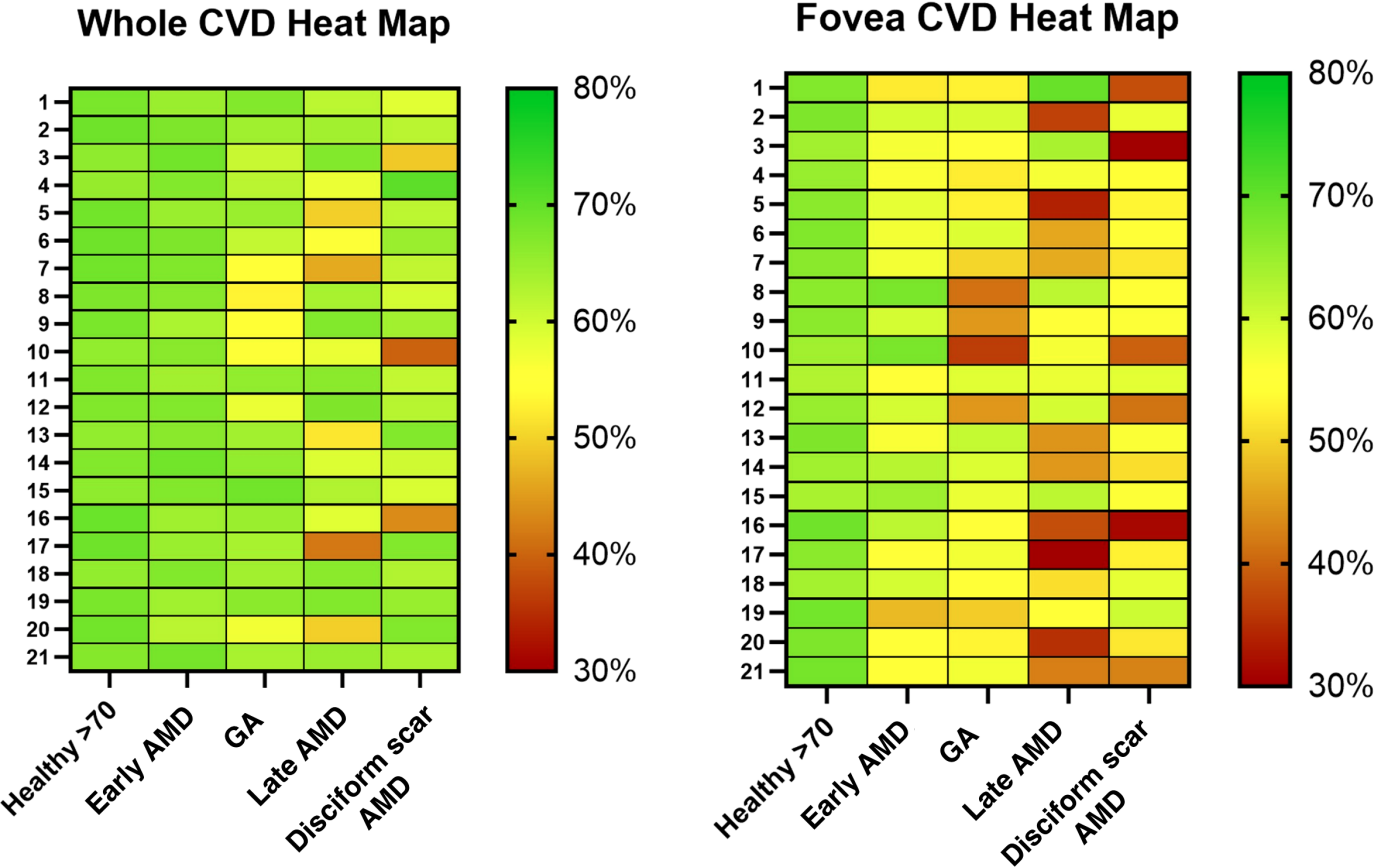
Heat maps of CVD for every eye highlighting variations in different colors (red for worse values, green for better). CVD, Choriocapillaris Vascular Density; GA, Geographic Atrophy; AMD, age related macular degeneration.

An overview on CVDs’ data among all subgroups is available in **Table 2**, whereas heat maps of the various CVD values are presented in **Figure 5**.

**Table 2:** Choriocapillaris vascular density among all sub-groups.

	CVD	Mean ± SD (%)	Range (%)	Mean difference	*P*
**Healthy over 70**	Whole	67.42 ± 1.34	65.4 - 69.3
	Fovea	66.12 ± 1.70	62.8 - 68.8
**Early AMD**	Whole	66,17 ± 1,77	62.2 - 68.7	1.26	0.86
	Fovea	58.32 ± 4.87	47,6 - 67,9	7.80	0.01*
**GA**	Whole	61.99 ± 4.65	52.9 - 68.7	5.44	0.005*
	Fovea	52.92 ± 6.56	36.3 - 61.0	13.20	<0.0001*
**Late AMD**	Whole	59.42 ± 7.67	41.8 - 67.4	8.00	<0.0001*
	Fovea	49.72 ± 11.43	27.2 - 69.6	16.40	<0.0001*
**Disciform scar AMD**	Whole	60.6 ± 7.64	39.8 - 70.5	6.83	0.0003*
	Fovea	49.05 ± 11.96	9.4 - 60.3	17.07	<0.0001*

Summary of CVD values in both whole macular and foveal areas among all subgroups, expressed in mean ± SD and in ranges from min to max. Mean differences in each subgroup are expressed in comparison to the Healthy control group. Asterisk refers to statistically significant differences in CVD among groups in analysis. CVD, Choriocapillaris Vascular Density; GA, Geographic Atrophy; AMD, age related macular degeneration.

### GA subgroup results

The GA subgroup underwent an extra analysis focused on the areas of inner/outer segment of photoreceptors (IS/OS) disruption, i.e., EZ interruption. An average of 1954.5 ± 1477.6 µm of EZ interruption was found on OCT B scans, while ImageJ analysis reported a mean atrophic area of 9.11 ± 9.22 mm^2^ on 6.4x6.4 mm macular scans.

In this subgroup, the *whole image*-CVD had an inverse correlation with both the EZ interruption measurements (r=-0.5390; R^2 =^ 0.31; p=0.012) and the calculated chorio-retinal atrophic area (r=-0.5546; R^2=^ 0.29; p=0.009). On the other hand, the *foveal* CVD did not correlate with any of them (r=-0.2112; p=0.36 for foveal CVD and EZ interruption correlation; r=-0.03925; p=0.87, for foveal CVD and measurement of the atrophic area).

### Other results

**Figure 6:**
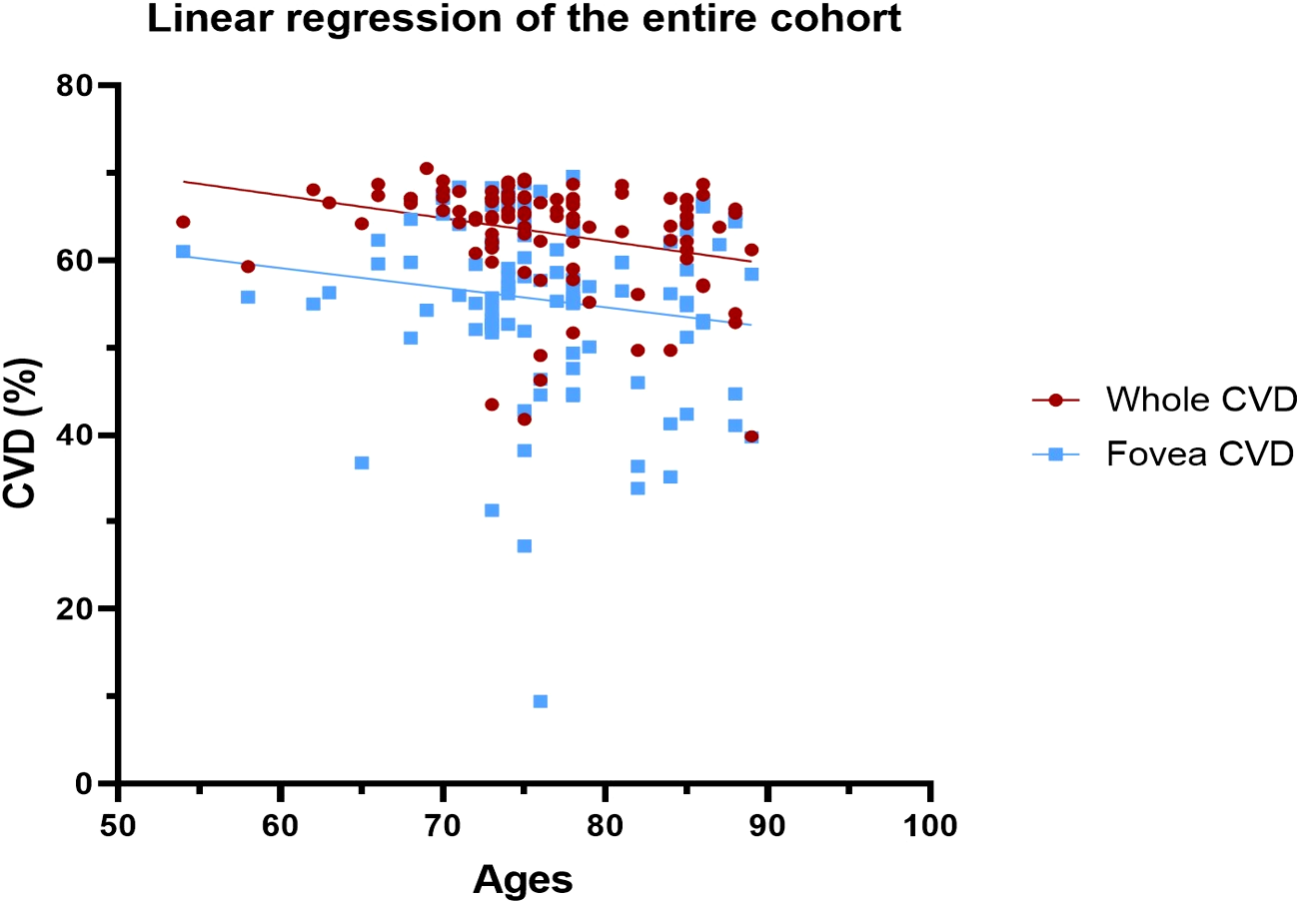
Linear regression to evaluate age-dependent CVD changes in both whole and foveal area among the entire cohort. CVD, Choriocapillaris Vascular Density.

Correlation analysis among all subgroups showed an inverse correlation between patients’ age and *whole image* CVD (r=-0.2875; R^2=^ 0.08; p=0-003). On the other hand, no correlation was found between patients’ age and *foveal* CVD (r=-0.1476; p=0.13). Linear regression analysis showed a significant non-zero slope when considering the entire sample and evaluating patients’ age and *whole* CVD (slope=-0.2612, 1/slope=-3.828; p=0.003), whereas this outcome was not appreciated when evaluating the *foveal* CVD (slope=-0.2242, 1/slope=-4.461; p=0.13) (**Figure 6**).

Finally, a *post hoc* analysis was conducted on healthy patients under 70 years old, with a mean age of 61.2 ± 8.2 years (N=21 eyes). The pairwise comparison between under-70 and over-70 years old healthy controls showed no statistically significant difference in either their *whole image* CVD (mean difference 0.86, p=0.30), or in their *foveal* CVD (mean difference 0.36, p=0.88). Correlation analysis showed a statistically significant inverse correlation between age and *whole image* CVD (r=-0.3844, p=0.012), and only a non-significant tendency to inverse correlation with *foveal* CVD (r=-0.2314, p=0.14). These results were confirmed with linear regression analysis.

## Discussion

Several papers have recently explored the role of CC flow in many types of age-related macular degeneration.

A considerable drop in CC perfusion has been demonstrated beneath (29, 30) and even beyond the boundaries of GA areas (31, 32). Particularly, Sacconi et al. found a reduction in vessel density at GA’s borders, and hypothesized that this impairment might forecast the future direction of its expansion (33). Nassisi et al., analyzing CC flow around zones of GA with SS-OCTA found that the 500-µm para-atrophic zone directly around the GA had much higher flow deficits (FDs) than the 500-µm peri-atrophic ring, i.e., just beyond the former (34). This result was confirmed by Rinella et al., who evidenced a significant increase in CC flow voids in GA areas, compared to age-matched controls, but also in the area within 2° of the GA, compared with the area outside this limit. These findings suggest that a proportional reduction of flow starts from the edges of the atrophy (35). Again, Nassisi et al. recently demonstrated that the severity of FDs around the GA is related to the speed with which the illness progresses or the atrophy enlarges (12, 34). Based on these findings, it could be hypothesized that CC flow modification may occur before RPE atrophy, making the CC alteration a biomarker of GA progression (36). However, the above mentioned results are in contrast with those of Bhutto et al., who suggest that the first insult begins at the level of the RPE, while the CC would be affected only afterwards (37). It was also hypothesized a strict connection between CC and RPE, necessarily through few soluble molecules since the presence of the Bruch’s membrane. A role seemed to be particularly played by the VEGF (vascular endothelial growth factor) and its receptor VEGFR2 (38).

Alagorie et al. focused on the severity of CC FDs in peripheral portions of the macula in patients with late AMD. They claimed that while the peripheral CC flow in eyes with CNV was comparable to that in healthy eyes, the FDs in GA-eyes was considerably higher in comparison with controls (p=0.012) and CNV eyes (p=0.038) (39). Consistent with these findings, on whole images (i.e., macular area), we found a significant inverse correlation between CVD changes and atrophic parameters (i.e., the size of macular chorio-retinal atrophy and the length of EZ interruption) in the GA subgroup.

Sohn et al. recently evidenced that CC loss can be detected in early AMD even in regions with a preserved RPE. On the other hand, modifications in the lumen/stroma ratio of the outer choroid, did not significantly vary between controls, AMD, or GA eyes, suggesting that CC changes are more common/happen earlier than those in the outer choroid (11). In contrast with this finding, another research group found no difference in CC blood flow between intermediate AMD patients and healthy control participants (40), and also in our study, no statistically significant difference in CVD values between healthy and early AMD eyes, was to be found. Interestingly, a previous research on intermediate AMD suggested that there are CC FDs below the drusen and in the areas immediately around them (41). Some authors believe that a compensatory enhanced CC perfusion in a more remote area of the macula, away from the drusen, might lead to an overall normal macular CVD (39).

In agreement with the above, we detected a progressive attenuation of the CVD as the AMD progressed to more advanced stages. A recent study conducted by Savastano et al. showed a statistically significant difference in CC perfusion in both the entire macular area and the foveal region between healthy and advanced exudative - AMD eyes, suggesting that a loss in CC perfusion should be interpreted as a marker of permanent damage, and thus a predictor of AMD evolution (42).

This research highlighted that there is a statistically significant difference in *whole images*-CVDs between various types of AMD eyes (with the exception of early AMD) and control groups. F*oveal* CVD, on the other hand, showed a significant difference between healthy controls and all stages of AMD. Moreover, comparison between the early AMD, late AMD and the disciform scar AMD groups showed a significant reduction in CVD, with increasing mean differences, in both *whole* and *foveal images*. This may suggest that a linear decrease of CC perfusion goes hand in hand with exudative AMD worsening and reflects the disease’s stage. Nonetheless, we found no differences in CVD between mild/intermediate forms of AMD and GA. Although not statistically significant, in our study CC flow impairment in GA atrophy tended to be less severe than in the advanced exudative forms of AMD.

Based on our findings, we hypothesize that the detection of decreased CVD on *foveal images* in patients at the early stage of AMD could represent a useful functional biomarker for an early diagnosis, since it seems to become evident before any morphological modification. This is consistent with a recent research conducted by Tiosano et al., in which a group of patients with a relatively stable intermediate AMD followed with OCTA, showed a significant CC impairment deterioration over one year of follow up, suggesting that CC dysfunction occurs before any structural change (43). Similarly, Corvi et al. suggested that CC flow deficit, along with other structural biomarkers such as intraretinal hyperreflective foci, hyporeflective drusen cores, and higher drusen volume ≥0.03 mm^3^, was an independent risk factor for the progression of intermediate AMD to outer retinal and RPE atrophy (44).

Our study has several limitations. First, because intraretinal fluid can mask the signal from the underlying choriocapillaris (i.e., absent flow signal), CVD might have been described as falsely reduced in case some lesions would have layed on top of the CC itself (i.e., “dark halo” effect). Indeed, previous studies have found an increase in the percentage of CC FDs of areas immediately surrounding CNVs (39, 45, 46). Another possible shortfall of our study derives from the manual segmentation of GA areas. However, the inverse correlation we have found between GA’s parameters and the CVD had an important statistical significance (p=0.012 and p=0.009), making a casual correlation unlikely.

In conclusion our study highlighted the crucial role that OCTA could play in the categorization of AMD. Indeed, this imaging technique is able to detect significant worsening in CVD among the different phases of the disease. Importantly, the detection of an early decrease in CVD may be a predictor of AMD development, becoming a fundamental biomarker of this pathology.

## Data availability statement

The raw data supporting the conclusions of this article will be made available by the authors, without undue reservation.

## Ethics statement

The studies involving human participants were reviewed and approved by Catholic University of the Sacred Heart Ethical Committee, Rome, Italy. The patients/participants provided their written informed consent to participate in this study.

## Authors contributions

Data collection: CFo, CFa, MMC Writing paper: MCS; CFo; MMC Idea of paper investigation: MCS. Data manager: CR; RK. Review: MCS; AS. Statistical Analysis: BF. Language assistance: RK. Critical revision: SR. All authors have read and agreed to the published version of the manuscript.

## Acknowledgments

The Authors want to thank Fondazione Policlinico Gemelli IRCCS and Ministero della Salute. The Authors want to thank Catholic University of the Sacred Heart, too. The Authors thank native speaker co-author Raphael Kilian for language assistance.

## Conflict of interest

The authors declare that the research was conducted in the absence of any commercial or financial relationships that could be construed as a potential conflict of interest.

## Publisher’s note

All claims expressed in this article are solely those of the authors and do not necessarily represent those of their affiliated organizations, or those of the publisher, the editors and the reviewers. Any product that may be evaluated in this article, or claim that may be made by its manufacturer, is not guaranteed or endorsed by the publisher.
